# Quantification of Contralateral Differences of the Scaphoid: A Comparison of Bone Geometry in Three Dimensions

**DOI:** 10.1155/2014/904275

**Published:** 2014-02-11

**Authors:** Claudio Letta, Andreas Schweizer, Philipp Fürnstahl

**Affiliations:** ^1^Department of Surgery, Spital Flawil, Kantonsspital St. Gallen, Krankenhausstrasse 23, 9230 Flawil, Switzerland; ^2^Department of Orthopaedic Surgery, University Hospital Balgrist, University of Zurich, Forchstrasse 340, 8008 Zurich, Switzerland; ^3^Computer Assisted Research & Development Group, University Hospital Balgrist, University of Zurich, Forchstrasse 340, 8008 Zurich, Switzerland

## Abstract

The purpose of this study was to accurately quantify contralateral differences of the scaphoid in three-dimensional space to evaluate the feasibility of using the healthy contralateral bone as a reconstruction template in the preoperative planning of complex mal- or nonunions. Three-dimensional surface models of the left and right scaphoids were reconstructed from computed tomography images and compared in 26 individuals. Left-right differences were quantified with respect to volume, surface area, length, and surface-to-surface deviation. The average left-right differences in volume, surface area, and length were 95.4 mm^3^ (SD 66.2 mm^3^), 32.7 mm^2^ (SD 22.9 mm^32^), and 0.28 mm (SD 0.4 mm), respectively. The average surface-to-surface deviation between the sides was 0.26 mm (SD 0.2 mm). High statistical correlation (Pearson) between the left and the right side was found in all evaluated measures.

## 1. Introduction

Scaphoid injury is the most common pathology of the carpal bones [1]. The reported incidence of nonunion after conservative treatment has been more than 12%; particularly, young men have the highest incident rate [1, 2]. Most common reasons for scaphoid nonunion are inadequate immobilization or displaced fragments [3]. An established nonunion [4–8] or malunion [9–13] after fracture can lead to pain, loss of function, and osteoarthritis. The preferred treatment option is surgical reduction with internal fixation due to its excellent fracture union rate of more than 95% [14, 15]. The aim of treatment should be bone healing with restoration of the scaphoid shape. However, the surgical procedure is challenging [3].

As a consequence, several authors emphasized the importance of preoperative planning [16], since the accuracy of the reduction is primarily dependent on the preoperative quantification [17, 18]. For the surgical correction of complex mal- or nonunions, the surgeon can use the opposite healthy bone as a guideline [11, 16, 18, 19]. Moreover, recently developed computer methods for the 3D preoperative planning of surgical reconstruction do also use the contralateral bone as a gold standard [18, 20]. For this reasons, the quantitative knowledge of contralateral differences in 3D may help to support the surgeon in the clinical problem. However, left-right variability was rarely evaluated and the exact knowledge about 3D differences is still limited [21].

This study aims at the exact quantification of contralateral differences of the scaphoid bone geometry using computer algorithms. The developed measurement techniques can be applied to 3D surface models in an automatic fashion to avoid inaccuracies due to manual measurements. Additional emphasis was put on quantifying surface-to-surface deviation between the sides, which is crucial for preoperative planning approaches based on the contralateral bone [18].

## 2. Materials and Methods

Between August 2009 and April 2012, bilateral computed tomography (CT) images had been acquired in 26 patients, who were treated with computer-assisted distal radius osteotomies. Based on these data, we analysed 52 healthy scaphoids (26 pairs) with respect to geometrical differences. Approval from the responsible ethics committee and informed patient consent was obtained for analysing this data in a retrospective way. The male-to-female ratio was 15 to 11 and the mean age of the patients was 32 years (SD 15.2 years, range 13–71 years). There was no evidence of scaphoid deformity due to previous trauma.

The image data was acquired using a Philips Brilliance 40 CT device (120 kV, Philips, Best, The Netherlands) with an axial resolution of 1.0 mm. For preprocessing, the DICOM files were imported into commercially available image processing software (Mimics, Materialise, Leuven, Belgium). The segmentation of the cortical bone layer was performed by applying manual thresholding, followed by region growing in order to separate the scaphoid from surrounding bones. In the last step, a 3D polygonal surface model of the outer bone contour was generated by applying the Marching Cubes algorithm [22].

Different measures were applied to the 3D models of the left and right scaphoids of each subject. Quantification of the geometry, based on orientation-independent features such as volume, surface area, and length, was performed to allow comparison with historical data. Additionally, the surface-to-surface deviation between the left and the right side was measured.

Methods for precisely computing the surface area and the volume of a 3D model are well known and straightforward. The surface area of the left (right) scaphoid was computed by summing up the area of each polygon (i.e., triangle) of the 3D model as described in [23].

Before calculating the volume of the surface model, it has to be described as a solid object. To this end, each triangle of the model was converted to an elementary tetrahedron, formed by the three vertices of the triangle and the origin of the coordinate system [23]. The volume of the model was finally determined by summing up the signed volumes of the elementary tetrahedra.

The scaphoid length was quantified using a 3D method not relying on manual measurements. Basically, the length can be determined by measuring the distance between the most proximal and most distal points on the scaphoid poles [24, 25]. In 3D, we propose to use the concept of the oriented minimum bounding box (OBB) as described in [26]. The OBB encloses all points while its length axis points in direction of the largest possible variance of the data. Therefore, the length (i.e., the largest dimension) of the OBB was used to describe scaphoid length.

In order to determine contralateral differences in surface, the left and right scaphoids were superimposed. To this end, the right scaphoid was mirrored and coarsely aligned to the contralateral bone. Thereafter, the iterative closest point (ICP) surface registration algorithm [27] was applied for accurate and automatic alignment, minimizing the point-to-point distances between the models in a least square sense. This technique was previously used in studies for quantifying scaphoid nonunion [18] as well as for comparing side-to-side differences of the tibia [28]. After registration, the surface-to-surface deviation was measured by comparing the distances between the outer contours of the models on a per-point basis. The mean surface-to-surface deviation (i.e., the root mean squared error RMSE) was defined as the average distance from the points of one model to the closest point on the other model.

Statistical analysis of the data was carried out using the software R (R Foundation, Vienna, Austria).

## 3. Results and Discussion

### 3.1. Results

**Figure 1:**
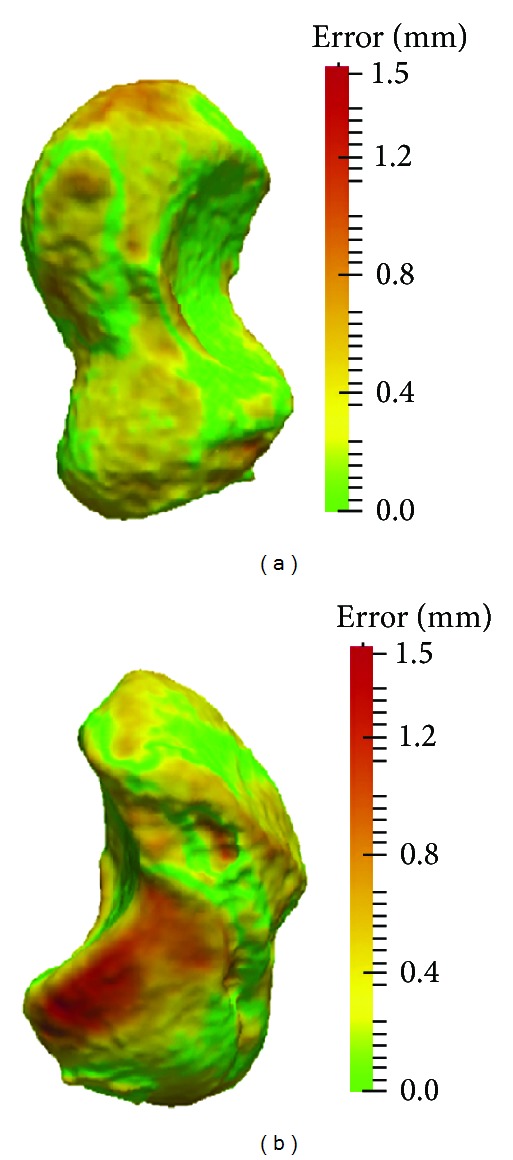
Scaphoid with the largest left-right surface distance error in all subjects. Left and right scaphoids are given.

**Figure 2:**
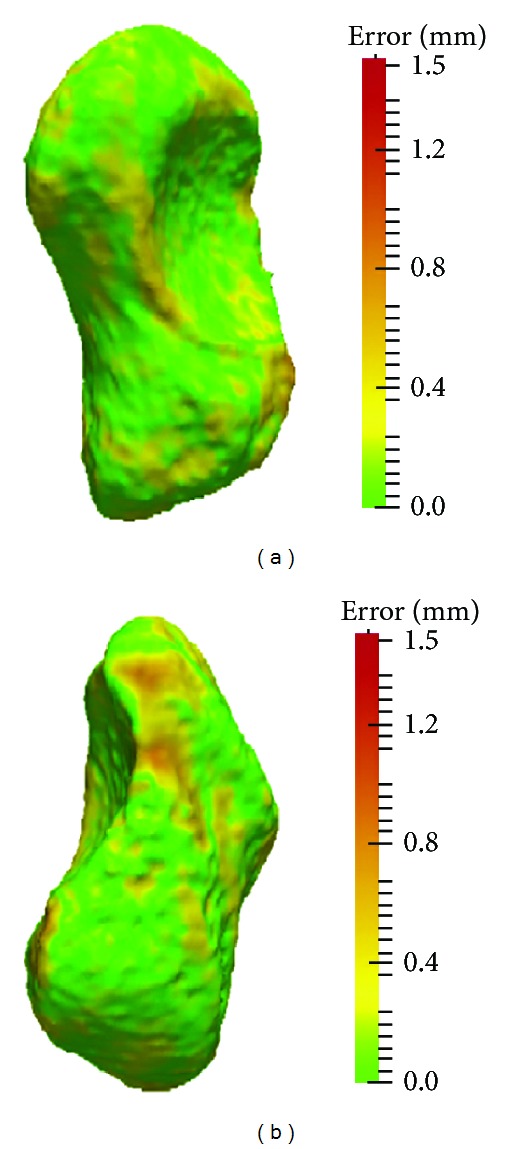
Scaphoid with the smallest left-right surface distance error in all subjects. Left and right scaphoids are given.

The results with respect to the proposed measures are given in Tables 1 and 2. The average left-right differences in volume, surface area, and scaphoid length among all subjects were 95 mm^3^ (SD 66 mm^3^, range: 4.8–235 mm^3^), 32 mm^2^ (SD 22 mm^2^, range: 0.7–84 mm^2^), and 0.5 mm (SD 0.4 mm, range: 0–1.4 mm), respectively. For these parameters, no significant side-to-side difference (Welch two sample *t*-test, *P* ≫ 0.05) between men (average difference in volume 101 mm^3^, area 34 mm^2^, and length 0.5 mm) and women (average difference in volume 87 mm^3^, area 30 mm^2^, and length 0.6 mm) could be observed. Contrary, the contralateral surface-to-surface deviation was significantly different (Welch Two Sample *t*-test, *P* < 0.05) between those groups with respect to both, the average and maximum left-right surface error. In men, the surface error was significantly higher (average 0.28 mm, SD 0.05 mm) as in women (average 0.22 mm, SD 0.05 mm). Figures 1 and 2 visualize the scaphoids having the largest (up to 1.9 mm) and smallest (up to 0.73 mm) surface deviations. On average, the surface error was 0.26 mm (SD 0.2 mm) in the 26 subjects. The correlation between the left and the right sides was very high (Pearson, *P* < 0.01, 2-tailed), being 0.99 for surface and volume and 0.98 for the other measures.

In addition to the contralateral evaluation, the data allowed us to compare the variability of the scaphoids between subjects. The average volume of the left and right scaphoid among all subjects was 2500.0 mm^3^ (SD 962.2 mm^3^, range: 748–4931 mm^3^) and 2518.6 mm^3^ (SD 987.4 mm^3^, range: 877–4986 mm^3^), respectively. The average surface area was 1097 mm^2^ (SD 286 mm^2^, range: 459–1728 mm^2^) for the left side and 1099 mm^2^ (SD 287 mm^2^, range: 513–1722 mm^2^) for the right side. On average, the length of the left and right scaphoids was 26.75 mm (SD 3.7 mm, range: 16–33.9 mm) and 26.85 mm (SD 3.9 mm, range: 16.2–34 mm).

The size of the scaphoid between genders differed significantly (Welch two sample *t*-test, *P* < 0.05) in volume, surface area, and length, considering only adult subjects. Male had a larger scaphoid volume (40% larger on average), surface area (20% larger on average), and length (10% larger on average) than female.

### 3.2. Discussion

Compson and colleagues [29] were one of the first who mentioned the importance of considering the 3D shape of the scaphoid for performing surgical treatment. They concluded that 2D radiological images were insufficient to describe the complex shape of the scaphoid. Therefore, 3D preoperative analysis is essential for appropriate treatment of scaphoid nonunion [17, 30] and the use of 3D methods for measuring surface deviation may help to identify important contralateral differences.

Several studies analysed the anthropometry, morphology, and geometry of carpal scaphoids [17, 24, 25, 29–32] with the purpose to improve treatment of scaphoid fractures [1, 2, 4, 6, 14, 15] and nonunions [5, 7–10, 12, 17, 18]. Consistent with our results, a significant difference in size and length between the genders was demonstrated [24, 25, 30, 32].

The dimension of the scaphoid, particularly its length, was determined manually in CT [25, 30], MRI [32], and cadavers [24]. The scaphoid length in our study (men, average 27.5 mm and SD 4.4 mm; women, average 25 mm and SD 2.5 mm) was similar to the study of Pichler et al. [25] (men, average 27.8 mm and SD 1.6 mm; women, average 24.5 mm and SD 1.6 mm), smaller than in the studies of Heinzelmann et al. [24] (men, average 31.3 mm and SD 2.1 mm; women, average 27.3 mm and SD 1.7 mm) and Patterson et al. [30] (men, average 29.2 mm and SD 3.75 mm; women, average 25.5 mm and SD 2.3 mm), and larger than in the evaluation of Smith [32] (men, average 26 mm and SD 1.9 mm; women, average 22.5 mm and SD 1.4 mm). Pichler et al. also used a 3D surface model as the geometric representation which may be an explanation for the similar results.

Patterson et al. estimated the surface area and volume by approximating the shape with a set of cylinders, manually created on the slices of the CT image. Their measured volume (men, average 2524 mm^3^, SD 846 mm^3^; woman, average 1675 mm^3^, SD 482 mm^3^) and surface area (men, average 1235 mm^2^, SD 277 mm^2^; woman, average 920 mm^2^, SD 178 mm^2^) correlate well with the volume (men, average 2879 mm^3^, SD 1116 mm^3^; woman, average 2042 mm^3^, SD 638 mm^3^) and surface area (men, average 1141 mm^2^, SD 324 mm^2^; woman, average 954 mm^2^, SD 204 mm^2^) of our study. Pichler et al. developed a different method to measure the scaphoid volume. The volume was approximated by a polyhedron consisting of 16 points which were selected according to anatomical landmarks. However, their results (men, average 4058 mm^3^, SD 741 mm^3^; woman, average 2846 mm^3^, SD 618 mm^3^) differed considerably from other findings.

Only some of these studies [21, 30, 32] performed a comparison between the left and the right scaphoids. Patterson et al. described a CT-based analysis of 35 adult wrists obtained from 21 cadavers and 14 patients. They did not observe any significant difference between the left and right scaphoids with respect to wrist dimension. Smith [32] validated left-right symmetry based on three biometric measurements (i.e., scaphoid length, proximal pole height, and sagittal interscaphoid angle), identified in ultrathin (0.7 mm slice thickness) MRI-scans of 30 healthy subjects. The contralateral differences of these measurements were minimal (below 0.8 mm and 3.3°), showing a left-right correlation coefficient of 0.92 to 0.98. Heinzelmann et al. studied 30 pairs of cadaveric scaphoids. They did not found any considerable side-to-side difference between the left and the right side with respect to proximal pole, distal pole, waist, or length measurements. In a cadaveric study of Ceri et al. [21], 200 scaphoid bones were used to assess 11 morphometric parameters for left-right comparison. The contralateral length difference was 0.1 mm on average. In contrast to the other studies, significant side-to-side differences were found in 4 morphometric features (i.e., circumference of the waist, base of the tubercle, width of the main sulcus, and the secondary height of the tubercle). These results roughly agree with our study, where the average maximum surface-to-surface deviation was 1.23 mm. However, the differences reported by Ceri et al. were considerably smaller than their intra- and interobserver variability of 1.3 mm and 1.8 mm, respectively.

The discussion of previous work showed that very few studies quantified contralateral differences of the scaphoid, measuring only basic parameters. The evaluations relied on manual measurements, which may bias small left-to-right differences due to observer variability. Contrary, computer-based algorithms were applied to different anatomy for measuring surface differences in a more comprehensive way [28]. Similar methods were also used for 3D preoperative planning of the scaphoid [17, 18] and forearm bones [33]. Therefore, we used surface registration algorithms to evaluate surface-to-surface deviation of the scaphoid, which is strength of our study. Another advantage of our approach is that the proposed methods work in an automated fashion. Moreover, we used algorithms for surface area and volume computation [23], which can be accurately applied to surface models, instead of approximating these measurements [25, 30]. Limitations of the study are the relatively small sample size and the fact that only the cortical bone layer was examined without consideration of potential differences due to cartilage.

## 4. Conclusions

The scaphoid has a twisted and bean-formed shape which makes the analysis of complex mal- and nonunions based on conventional radiographs difficult. State-of-the-art computer-assisted surgical planning approaches rely on the contralateral scaphoid as a 3D reconstruction template. The goal of this study was to precisely quantify potential left-right differences with 3D techniques without estimating measurements. The evaluation of surface-to-surface differences revealed that regions of the scaphoid may differ up to 1.9 mm. This fact must be taken into account if the contralateral scaphoid should be used in surgical planning. Furthermore, the surface-to-surface deviation between men was significantly higher than in women. As in previous studies, high correlation between the left and right scaphoids with respect to volume, surface area, and length was observed.

## Figures and Tables

**Table 1:** Quantitative evaluation of the 3D scaphoid geometry.

	Volume L (mm³)	Volume R (mm³)	Area L (mm²)	Area R (mm²)	Length L (mm)	Length R (mm)
Woman	2021 (SD 602), 1146–3131	2041 (SD 645), 1133–3364	951 (SD 193), 654–1307	960 (SD 205), 658–1374	25 (SD 2.3), 21.3–29.4	25.2 (SD 2.8), 21.2–30.8

Men	2851 (SD 1040), 749–4932	2869 (SD 1064), 878–4986	1205 (SD 11), 460–1729	1201 (SD 312), 514–1722	28 (SD 4.5), 16–33.9	28 (SD 4.4), 16.2–34

Both	2500 (SD 262), 749–4932	2519 (SD 987), 878–4986	1097 (SD 292), 460–1729	1099 (SD 293), 514–1722	26.8 (SD 4), 16–33.9	26.9 (SD 4), 16.2–34

Average, standard deviation (SD), and range in volume, surface area, and maximal length of the left (L) and right (R) scaphoids are given for woman (first row), men (second row), and for all subjects (third row). SD denotes the standard deviation.

**Table 2:** Contralateral differences of the 3D scaphoid geometry.

	Volume L, R diff (mm³)	Area L, R diff (mm²)	Length L, R diff (mm²)	Average surface deviation (mm)
Woman	87.4 (SD 74.7), 10.2–235.1	30.2 (SD 21.9), 1.6–67.2	0.6 (SD 0.5), 0–1.4	0.22 (SD 0.04), 0–1.84

Men	101.1 (SD 61.4), 4.8–234.1	34.5 (SD 24.3), 0.7–85	0.5 (SD 0.4), 0–1.4	0.28 (SD 0.06), 0–1.98

Both	95.3 (SD 66.2), 4.8–235.1	32.7 (SD 22.9), 0.7–85	0.2 (SD 0.4), 0–1.4	0.26 (SD 0.2), 0–1.98

Average contralateral difference, standard deviation (SD), and range in volume, surface area, and maximal length between the left (L) and right (R) scaphoids are given for woman (first row), men (second row), and for all subjects (third row).
